# Biophysical characterization data of the artificial protein Octarellin V.1 and binding test with its X-ray helpers

**DOI:** 10.1016/j.dib.2016.07.036

**Published:** 2016-07-26

**Authors:** Maximiliano Figueroa, Julie Vandenameele, Erik Goormaghtigh, Marie Valerio-Lepiniec, Philippe Minard, André Matagne, Cécile Van de Weerdt

**Affiliations:** a GIGA-Research, Molecular Biomimetics and Protein Engineering, University of Liège, Liège, Belgium; b Biochemistry and Molecular Biology Department, University of Concepcion, Concepción, Chile; c Laboratoire d’Enzymologie et Repliement des Protéines, Centre for Protein Engineering, University of Liège, Liège, Belgium; d Laboratory for the Structure and Function of Biological Membranes, Center for Structural Biology and Bioinformatics, Université Libre de Bruxelles, Brussels, Belgium; e Institute for Integrative Biology of the Cell (I2BC), UMT 9198, CEA, CNRS, Université Paris-Sud, Orsay, France

**Keywords:** Artificial proteins, Circular dichroism, Crystallization helpers, Infra red spectroscopy, Protein design, Isothermal Titration Calorimetry

## Abstract

The artificial protein Octarellin V.1 (http://dx.doi.org/10.1016/j.jsb.2016.05.004[1]) was obtained through a direct evolution process over the *de novo* designed Octarellin V (http://dx.doi.org/10.1016/S0022-2836(02)01206-8[2]). The protein has been characterized by circular dichroism and fluorescence techniques, in order to obtain data related to its thermo and chemical stability. Moreover, the data for the secondary structure content studied by circular dichroism and infra red techniques is reported for the Octarellin V and V.1. Two crystallization helpers, nanobodies (http://dx.doi.org/10.1038/nprot.2014.039[3]) and αRep (http://dx.doi.org/10.1016/j.jmb.2010.09.048[4]), have been used to create stable complexes. Here we present the data obtained of the binding characterization of the Octarellin V.1 with the crystallization helpers by isothermal titration calorimetry.

**Specifications table**

**Table:** 

Subject area	*Biology, chemistry*
More specific subject area	*Structural biology*
Type of data	*Figures*
How data was acquired	*Fluorescence was measured in a Varian Cary Eclipse spectrofluorimeter; circular dichroism data was obtained on a Jasco-810 spectropolarimeter; infra red data was obtained on a Bruker IFS55 FTIR spectrophotometer; and the ITC data was obtained on an ITC200 microcalorimeter.*
Data format	*Analyzed*
Experimental factors	*All the experiments were performed with fresh produced and purified proteins*
Experimental features	*Stability and native-like features of the artificial protein Octarellin V.1 have been tested by biophysical characterization.*
Data source location	*Liège, Belgium*
Data accessibility	*Data is within this article*

## Value of the data

• The data can be used to show that a directed evolution process does not alter the secondary structure of a protein.
• The data can be used to demonstrate that large artificial proteins can be thermostable.
• The data can be applied to show the formation of stable complexes between an artificial protein and crystallization helpers (nanobodies and αRep).

## 1 Data

**Fig. 1:**
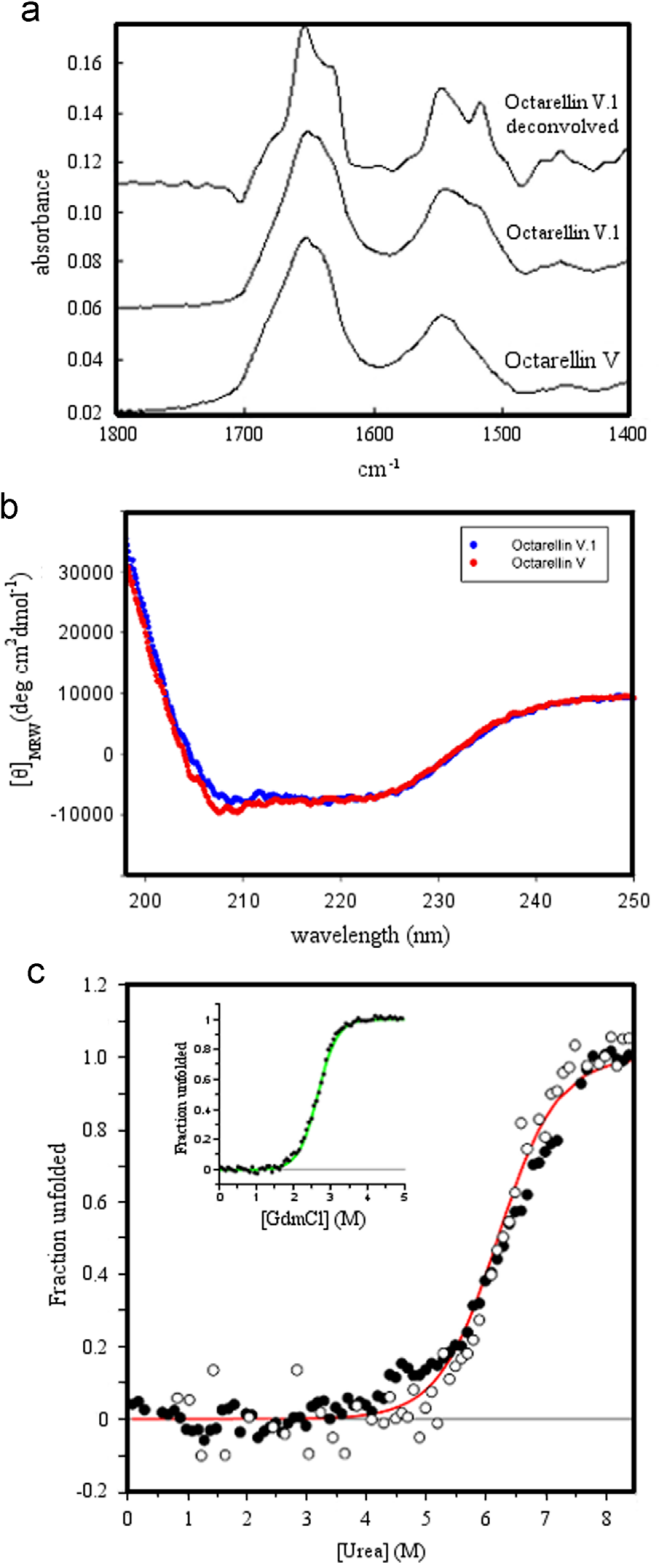
Biophysical characterization of Octarellin V.1. A) Infrared spectrum of Octarellin V and Octarellin V.1 recorded between 1800 and 1400 cm^−1^. Secondary structure was evaluated for both Octarellin V and Octarellin V.1. The determined helix contents were respectively 28% and 30% (standard deviation in cross-validation: 5.7%) and the sheet contents respectively 17% and 16% (SD: 6.7%). B) Far-UV CD spectra of Octarellins V and V.1, were recorded at 25 °C, in 50 mM phosphate buffer, pH 8.0, with protein concentration of 4.2 mM. The structural content of Octarellin V.1 was calculated from its spectrum, over the wavelength range of 250–190 nm. The results are: ~32%, ~22%, ~19%, and ~26% for helices, strands, turns, and unordered structures, respectively, with no significant structural differences as compared to Octarellin V. C) Urea-induced equilibrium unfolding transition of Octarellin V.1 at pH 8, 25 °C, monitored by the change in fluorescence intensity at 335 nm (○) and the change in ellipticity at 222 nm (•). Data were analyzed on the basis of a two-state model and the solid line was drawn using ∆*G*_NU_=31 kJ mol^−1^ and *m*_NU_=−5 kJ mol^−1^ M^−1^. The inset shows the GdmCl unfolding transition as obtained by far UV-CD at 222 nm, with the solid line drawn using ∆*G*_NU_=26 kJ mol^−1^ and *m*_NU_=−9.7 kJ mol^−1^ M^−1^. All data are presented as the fractional change in signal as a function of denaturant concentration.

**Fig. 2:**
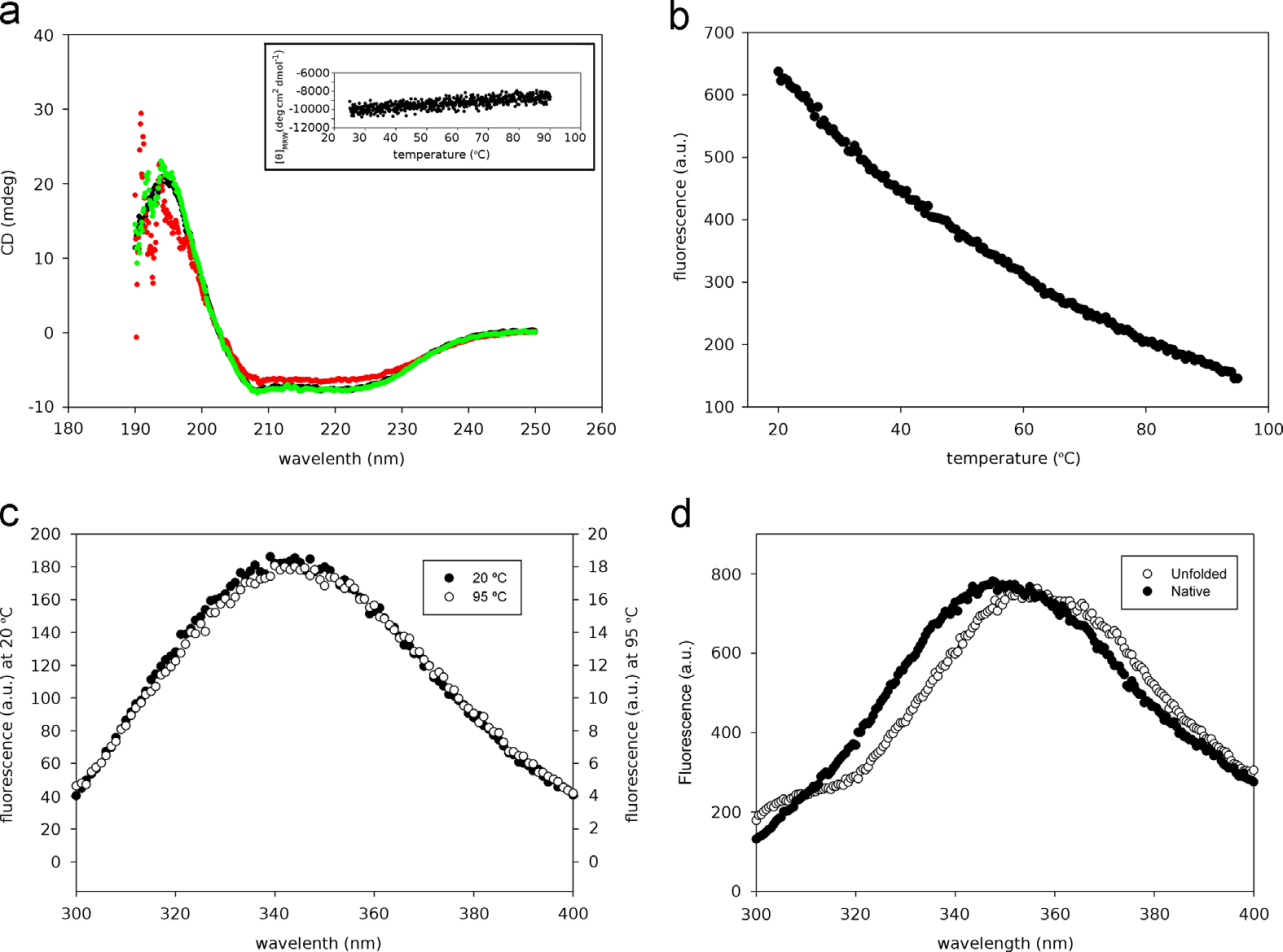
Octarellin V.1 shows no heat-induced denaturation. A) Far-UV CD spectra of Octarellin V.1 at 25 °C (black), 90 °C (red), and after cooling from 90 to 25 °C (green). A melting curve in the range of 25–90 °C, obtained by monitoring the ellipticity at 222 nm, is shown as an insert. B) Melting curve in the range 20–95 °C followed by tryptophan fluorescence. No structural transition is observed and the decrease in the signal intensity is merely due to heat-induced quenching. To corroborate this observation, the comparison of fluorescence spectra recorded at 20 °C and 95 °C (panel C) shows no shift in *λ*_max_, indicating no significant change in the environment of indole tryptophan side chain. D) Redshift of the fluorescence emission spectrum following chemically-induced denaturation of Octarellin V.1 in presence of 5.5 M GdmCl.

**Fig. 3:**
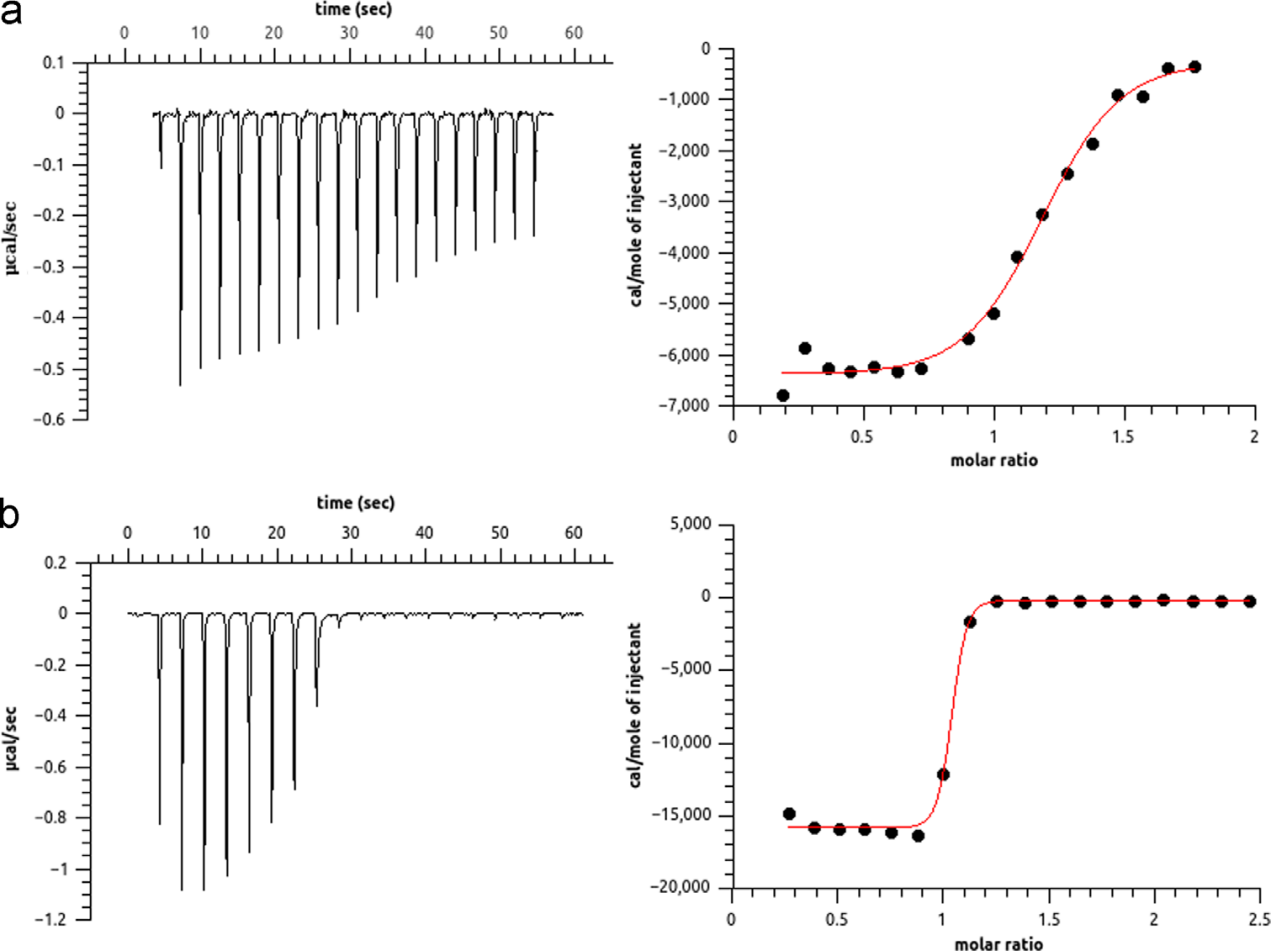
Characterization of the protein complexes nanobody/Octarellin V.1 and aRep/Octarellin V.1 by isothermal titration calorimetry (ITC). A) αRep/Octarellin V.1 complex. ITC characterization showed a Kd=0.45 μM and 1:1 stoichiometry (*N*=1.16). B) Nanobody/Octarellin V.1 complex. ITC characterization showed a Kd=15.09 nM and again 1:1 stoichiometry (*N*=0.979).

The data shared in this article is the biophysical characterization of the artificial protein Octarellin V.1 [1]. Using circular dichroism and infra red techniques we have compared the secondary structure profile of the parental Octarellin V [2] with the evolved Octarellin V.1 (Fig. 1A) and B)). The chemical stability of the Octarellin V.1 protein is presented as well in Fig. 1C). Moreover, by circular dichroism and fluorescence techniques we show the thermostability of this protein (Fig. 2). Finally, we present the Isothermal Titration Calorimetry (ITC) data (Fig. 3) for the interaction between Octarellin V.1 and two different crystallization helpers, nanobodies [3] and αRep [4].

## 2 Experimental design, materials and methods

### 2.1 Biophysical characterization

#### 2.1.1 ATR-FTIR spectroscopy

Attenuated total reflection infrared (ATR-FTIR) spectra were obtained on a Bruker IFS55 FTIR spectrophotometer (Ettlingen, Germany) equipped with an MCT detector (broad band 12,000-420 cm^−1^) at 2 cm^−1^ resolution with a 3.5-mm aperture. Each corrected spectrum was smoothed by apodisation of its Fourier transform by the Fourier transform of a 4-cm^−1^ Gaussian line. Fourier self deconvolution was performed according to Kauppinen et al. [5].

#### 2.1.2 Circular dichroism measurements

Circular dichroism (CD) measurements in the far-UV region (190–250 nm) were performed with a Jasco J-810 spectropolarimeter at 25 °C, in 50 mM phosphate buffer, pH 8.0. Protein concentrations were 0.1 mg mL^−1^ and 0.1 cm pathlength. Four scans were averaged, and base lines were subtracted. Secondary structure analyses using the CDSSTR, CONTINLL, and SELCON3 algorithms were performed on the CD data with the Dichroweb [6] analysis server.

#### 2.1.3 Denaturant-induced unfolding transitions

Samples at various GdmCl concentrations (0–5.5 M) or urea (0–8.5 M) were left to equilibrate for at least 18 h. Unfolding curves were determined by monitoring the changes in intrinsic fluorescence emission (*λ*_ex_=280 nm and *λ*_em_=335 (GdmCl) or 370 (urea) nm) and CD at 222 nm, at 25 °C. A protein concentration of 0.1 mg/mL (~4.3 μM) was used for both fluorescence and CD measurements.

### 2.2 Thermostability assays

#### 2.2.1 Intrinsic fluorescence measurements

Emission spectra (excitation at 280 nm) were recorded on a Varian Cary Eclipse spectrofluorimeter equipped with a Peltier-controlled cell holder. 5 emission spectra were recorded in the 300–420 nm range, using 1-cm pathlength cell and protein concentrations were 0.1 mg mL^−1^ in 50 mM phosphate buffer, pH 8.0, at 20 or 95 °C. A melting curve was obtained following the emission at 335 nm in the range 20–95 °C.

#### 2.2.2 Circular dichroism

Spectra were recorded as it was described before at 25 °C and 90 °C. Moreover, a melting curve was measured following the ellipticity at 222 nm in the range 25–90 °C.

#### 2.2.3 Isothermal titration calorimetry

The binding parameters were monitored with an ITC 200 microcalorimeter (MicroCal). For the titration of target protein, 2 μL aliquots of the titrant αRep or nanobodies (216 μM or 280 μM respectively) were injected from a computer-controlled 40 μL microsyringe at intervals of 180 s into the solution of Octarellin V.1 (25 μM or 30 μM respectively; cell volume 0.24 mL) dissolved in the same buffer. Analysis of the data was performed using the MicroCal Origin software provided by the manufacturer according to the one-binding-site model.
