# Supplementary material for: Biophysical characterization data of the artificial protein Octarellin V.1 and binding test with its X-ray helpers
Source: Data Brief. 2016 Jul 26;8:1221–6. doi: 10.1016/j.dib.2016.07.036 (PMC4982917; doi:10.1016/j.dib.2016.07.036)

Conflict of interest statement

All the authors of this manuscript certify that they have NO affiliations with or involvement in any organization or entity with any financial interest (such as honoraria; educational grants; participation in speakers' bureaus; membership, employment, consultancies, stock ownership, or other equity interest; and expert testimony or patent-licensing arrangements), or non-financial interest (such as personal or professional relationships, affiliations, knowledge or beliefs) in the subject matter or materials discussed in this manuscript.

I, Dr Maximiliano Figueroa, corresponding author of this work, sign in the name of all the authors.

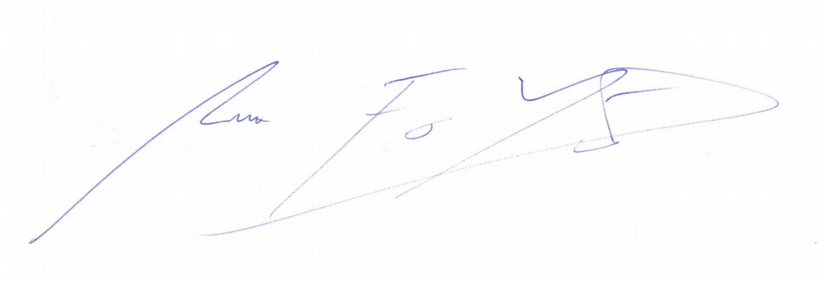

Supplement: Supplementary file 1 — Supplementary material [file mmc1.pdf]
